# Identifying Small Molecule-miRNA Associations Based on Credible Negative Sample Selection and Random Walk

**DOI:** 10.3389/fbioe.2020.00131

**Published:** 2020-03-17

**Authors:** Fuxing Liu, Lihong Peng, Geng Tian, Jialiang Yang, Hui Chen, Qi Hu, Xiaojun Liu, Liqian Zhou

**Affiliations:** ^1^School of Computer Science, Hunan University of Technology, Zhuzhou, China; ^2^Geneis (Beijing) Co. Ltd., Beijing, China; ^3^College of Chemical Engineering, Xiangtan University, Xiangtan, China; ^4^Xiangya Second Hospital, Central South University, Changsha, Hunan, China

**Keywords:** SMiR associations, random walk, negative sample selection, triple-layer heterogeneous network, drug repositioning

## Abstract

Recently, many studies have demonstrated that microRNAs (miRNAs) are new small molecule drug targets. Identifying small molecule-miRNA associations (SMiRs) plays an important role in finding new clues for various human disease therapy. Wet experiments can discover credible SMiR associations; however, this is a costly and time-consuming process. Computational models have therefore been developed to uncover possible SMiR associations. In this study, we designed a new SMiR association prediction model, RWNS. RWNS integrates various biological information, credible negative sample selections, and random walk on a triple-layer heterogeneous network into a unified framework. It includes three procedures: similarity computation, negative sample selection, and SMiR association prediction based on random walk on the constructed small molecule-disease-miRNA association network. To evaluate the performance of RWNS, we used leave-one-out cross-validation (LOOCV) and 5-fold cross validation to compare RWNS with two state-of-the-art SMiR association methods, namely, TLHNSMMA and SMiR-NBI. Experimental results showed that RWNS obtained an AUC value of 0.9829 under LOOCV and 0.9916 under 5-fold cross validation on the SM2miR1 dataset, and it obtained an AUC value of 0.8938 under LOOCV and 0.9899 under 5-fold cross validation on the SM2miR2 dataset. More importantly, RWNS successfully captured 9, 17, and 37 SMiR associations validated by experiments among the predicted top 10, 20, and 50 SMiR candidates with the highest scores, respectively. We inferred that enoxacin and decitabine are associated with mir-21 and mir-155, respectively. Therefore, RWNS can be a powerful tool for SMiR association prediction.

## 1. Introduction

miRNA is a small non-coding RNA molecule found in human beings, animals, plants, and even viruses (Bartel, 2004; Borges and Martienssen, 2015; Gebert and MacRae, 2019; Zhang et al., 2019b). miRNA can regulate gene expression and influence basic cellular functions, including proliferation, differentiation, and death (Lu et al., 2005; Gong et al., 2019). Overexpression and misregulation of miRNAs can result in great regulatory upheavals in the cell (Lu et al., 2005; Croce, 2009; Shigemizu et al., 2019) and produce phenotypes of human disease states (Trang et al., 2009; Chen et al., 2017a). For example, miR-21 is a well-known oncogenic miRNA, and its overexpression may result in onset of a variety of cancers, including ovarian, breast, lung, and gastric cancers (Esteller, 2011; Simonian et al., 2018). In Gastric Cancer (GC), its upregulation may lead to the suppression of tumor-suppressor genes, including PTEN, RECK, and PDCD4 (Kim et al., 2013), and promote proliferation, migration, and apoptosis inhibition (Zhang et al., 2008). Although miRNAs were discovered in the early 1990s (Lee et al., 1993; Wightman et al., 1993), related research did not achieve further progress until the 2000s (Reinhart et al., 2000; Lau et al., 2001). Many research studies have suggested that miRNAs play important roles in controlling many severe diseases, and miRNA can associate with diseases. Many computational models have been proposed to mine associations between miRNA and disease, such as AMVML (Liang et al., 2019), LPLNS (Li et al., 2018), and GRNMF (Xiao et al., 2017). Most drugs are composed of small molecules with a low molecular weight(<900 Daltons) (Huangfu et al., 2008). Small molecule drugs can regulate numerous cellular processes and thus heal diverse complex diseases (Lamb et al., 2006; Warner et al., 2018; Zhang et al., 2019a). More importantly, small molecules can inhibit miRNA pathways and regulate the metabolisms of humans (Sonnenburg and Bäckhed, 2016). New clues have been provided for various human disease therapies, including immune disorders and cancers, based on small molecules targeting miRNAs (Sevignani et al., 2006; Zhang et al., 2010; Abba et al., 2017; De Santi et al., 2017). For example, small molecules can inhibit the expression of miR-21 to activate tumor-suppressor genes by targeting miR-21 (Masoudi et al., 2018). Therefore, it has become a new therapy for human diseases to find miRNAs interacting with small molecules. Wet experiments discovered several Small Molecule drug-miRNA (SMiR) associations (Qu et al., 2018; Chen et al., 2020); however, this is a costly and time-consuming process. Therefore, various computational models are currently being explored to uncover potential SMiR associations based on small molecule similarity, the disease phenotype similarity of miRNA, and the SMiR association network (Monroig et al., 2015; Chen et al., 2020). Lv et al. (2011) and Qu et al. (2018) proposed SMiR association models based on random walk with restart. Jiang et al. (2012) identified new SMiR associations based on the expression difference of miRNA target genes and therapy drugs from 17 different cancers. Meng et al. (2014) explored a systematic computational model (smiRN-AD) to construct a bioactive SMiR association Network. smiRN-AD integrated gene expression data from bioactive small molecule perturbation and Alzheimer's disease-related miRNA regulation. Li et al. (2016) designed a network-based miRNA pharmacogenomic model, SMiR-NBI, integrating relevant biological information, including drugs, miRNAs, genes, and a network-based inference approach into a unified framework. SMiR-NBI effectively discovered potential response mechanisms of anticancer drugs targeting miRNAs and found that miRNAs may be underlying pharmacogenomic biomarkers in cancers. Chen et al. (2017b) developed an NRDTD database. NRDTD provides 165 non-coding RNA-drug associations supported by wet and clinical experiments from 96 drugs and 97 non-coding RNAs. Wang et al. (2019) developed a random forest-based SMiR prediction model, RFSMMA. Zhao et al. (2020) found SMiR association candidates based on symmetric non-negative matrix factorization and Kronecker regularized least squares. Yin et al. (2019) discovered underlying SMiR association-based sparse learning and heterogeneous graph inference. Qu et al. (2019) identified possible SMiR associations based on the HeteSim algorithm. These methods effectively improved SMiR association prediction performances. However, no negative samples (non-associating SMiR pairs) were available for validation. Therefore, these models had to randomly select parts of unobserved small molecule-miRNA pairs (unlabeled samples) as negative samples. However, these extracted negative samples probably contained positive SMiR associations, and this thus severely affects the prediction performance of computational models. More importantly, some methods, for example, TLHNSMMA (Qu et al., 2018), require numerous computational resources. Inspired by graph embedding methods on biomedical networks (Yue et al., 2020), we developed a new SMiR association prediction model, RWNS, integrating credible negative sample selection, random walk with restart, and diverse biological information into a unified framework. It includes three procedures: similarity computation, negative sample selection, and SMiR association prediction based on random walk with restart on the constructed small molecule-disease-miRNA association network (triple-layer network). RWNS computed small molecule similarity based on side effects, chemical structures, disease phenotypes, and gene functional consistency and miRNA similarity based on disease phenotypes and gene functional consistency. RWNS selected highly credible negative SMiR associations based on obtained similarity information. RWNS then iteratively performed a random walk with restart on the constructed triple-layer heterogeneous network to propagate association information and discover SMiR candidates. To evaluate the performance of RWNS, we used leave-one-out cross-validation (LOOCV) and 5-fold cross validation to compare RWNS with two state-of-the-art SMiR association methods, namely, TLHNSMMA and SMiR-NBI. Experimental results showed that RWNS obtained better improvement, and enoxacin and decitabine may be associated with mir-21 and mir-155, respectively. Therefore, RWNS could be a powerful tool for SMiR association prediction.

## 2. Materials and Methods

### 2.1. Small Molecule-miRNA Associations

The SMiR association network was obtained from the SM2miRdatabase (Liu et al., 2012). There are 664 experimentally validated SMiR associations in the database. Two datasets were applied to compare the performance of RWNS with two state-of-the-art methods, TLHNSMMA and SMiR-NBI. Dataset 1 (SM2miR1) contained 831 small molecules and 541 miRNAs. Dataset 2 (SM2miR2) contained 39 small molecules and 286 miRNAs. Only a part of the small molecules and miRNAs were involved in the known 664 SMiR associations from the SM2miRdatabase in dataset 1; however, all small molecules and miRNAs were fully involved in the known 664 SMiR associations in dataset 2.

An adjacency matrix ***M***_***sm***_ was used to indicate the known SMiR associations. The value of ***M***_***sm***_(*i,j*) was 1/664 if a small molecule *s*(*i*) interacted with an miRNA *m*(*j*), and otherwise it was 0. Furthermore, variables *s* and *m* were defined as the number of small molecules and miRNAs, respectively.

(1) $$\begin{array}{l} M_{sm}(s(i),m(j))=\{\begin{array}{cc} 1/664 & \text{s}(\text{i})\text{ is related to m}(\text{j})\\ 0 & otherwise \end{array} \end{array}$$

### 2.2. Human miRNA-Disease Associations

Human miRNA-disease association data was obtained from the HMDD database (v2.0) (Li et al., 2013). We performed the same preprocessing as TLHNSMMA and deleted disease-related miRNAs that were not involved in the known 664 SMiR associations. As a result, we downloaded 6,233 miRNA-disease interactions and constructed an adjacency matrix ***M***_*md*_ to indicate miRNA-disease associations. The value of ***M***_*md*_(*i,j*) was 1/6, 233 if an miRNA *m*(*i*) interacted with a disease *d*(*j*), and otherwise it was 0. Variables *m* and *d* were defined as the number of miRNAs and diseases, respectively.

(2) $$\begin{array}{l} M_{md}(d(i),m(j))=\{\begin{array}{cc} 1/6233 & d(\text{i})\text{ is related to m}(\text{j})\\ 0 & otherwise \end{array} \end{array}$$

### 2.3. Small Molecule Similarity

#### 2.3.1. Side Effect Similarity

We downloaded side-effect information on small molecules from the SIDER database (Kuhn et al., 2010). Two small molecules are more similar if they share more side effects based on guilt-by-association. The similarity value is 0 if two small molecules do not share any side effects. Suppose that *N*(*i*) represents a side effect set related to a small molecule *s*(*i*); ${SM}_{s}^{side}\left(i,j\right)$ indicates side effect similarity between *sm*(*i*) and *sm*(*j*). We computed side-effect similarity of small molecules based on the Jaccard formula via Equation (3). |***X***| represents the cardinality of set ***X***.

(3) $$\begin{array}{l} {SM}_{s}^{side}\left(sm\left(i\right),sm\left(j\right)\right)=Jaccard=\frac{\left|N\left(i\right)\cap N\left(j\right)\right|}{\left|N\left(i\right)\cup N\left(j\right)\right|} \end{array}$$

#### 2.3.2. Chemical Structure Similarity

SIMCOMP (Hattori et al., 2003) (http://www.genome.jp/tools/simcomp) is a graph-based tool that can be used to compute small molecule similarity based on chemical structures extracted from the COMPOUND and DRUG sections of the KEGG LIGAND database (Kanehisa et al., 2012). We used the tool to search a maximal share sub-graph isomorphism between small molecules *sm*(*i*) and *sm*(*j*) and computed their chemical structure similarity ${SM}_{s}^{ch}\left(i,j\right)$.

#### 2.3.3. Disease Phenotype-Based Similarity

We extracted small molecule-related diseases from Comparative Toxicogenomics Database (CTD) (Davis et al., 2013), DrugBank (Kuhn et al., 2010), and Therapeutic Targets database (TTD) (Zhu et al., 2011). Based on the assumption that two small molecules are more similar if they share more diseases, disease phenotype-based similarity $SM_{s}^{dis}\left(i,j\right)$ between small molecules *sm*(*i*) and *sm*(*j*) can be computed via Equation (4).

(4) $$\begin{array}{l} {SM}_{s}^{dis}\left(sm\left(i\right),sm\left(j\right)\right)=\frac{\left|S\left(i\right)\cap S\left(j\right)\right|}{\left|S\left(i\right)\cup S\left(j\right)\right|} \end{array}$$

#### 2.3.4. Gene Functional Consistency-Based Similarity

We extracted target genes of small molecules from DrugBank (Law et al., 2013) and TTD (Li et al., 2017). Based on the assumption that two target genes tend to be more similar if they share more functional consistency, we can compute functional consistency-based similarity ${SM}_{s}^{tar}\left(i,j\right)$ between two small molecules *sm*(*i*) and *sm*(*j*) via the Gene Set Functional Similarity (GSFS) method provided by Lv et al. (2011).

#### 2.3.5. Fused Small Molecule Similarity

We designed a weighted combination technique to fuse small molecule side effects, chemical structures, gene functions, and diseases phenotypes. The weighted combination technique can decrease the deviation of each separated similarity and balance the four different similarities. The fused small molecule similarity ***SM***_***s***_ can defined as shown via Equation (5).

(5) $$\begin{array}{l} SM=\left(\delta_{1}{SM}_{s}^{side}+\delta_{2}{SM}_{s}^{ch}+\delta_{3}{SM}_{s}^{dis}+\delta_{4}{SM}_{s}^{tar}\right)/\overset{4}{\underset{i}{\sum }}\delta_{i}\left(i=1,2,3,4\right) \end{array}$$

Here, the default value δ_*i*_ = 1 indicates that the four different similarities have the same weight.

### 2.4. miRNA Similarity

#### 2.4.1. Disease Phenotype-Based Similarity

We extracted miRNA-related diseases from HMDD v2.0 (Li et al., 2013), miR2Disease (Jiang et al., 2008), and PhenomiR (Ruepp et al., 2010). Based on the assumption that two miRNAs are more similar if they share more diseases, we could compute the disease phenotype-based similarity of miRNAs by using the Jaccard equation. Suppose that *M*(*i*) indicates the miRNA *m*(*i*)-related disease set. The disease phenotype-based similarity ${MR}_{s}^{dis}\left(i,j\right)$ between two miRNAs *mir*(*i*) and *mir*(*j*) can be calculated via Equation (6).

(6) $$\begin{array}{l} {MR}_{s}^{dis}\left(mir\left(i\right),mir\left(j\right)\right)=\frac{\left|M\left(i\right)\cap M\left(j\right)\right|}{\left|M\left(i\right)\cup M\left(j\right)\right|} \end{array}$$

#### 2.4.2. Gene Functional Consistency-Based Similarity

We extracted the target genes of miRNA from the TargetScan database (Friedman et al., 2009), and we calculated the functional consistency-based similarity ${MR}_{s}^{tar}\left(mir\left(i\right),mir\left(j\right)\right)$ between two miRNAs *mir*(*i*) and *mir*(*j*) based on GSFS (Lv et al., 2011).

#### 2.4.3. Fused miRNA Similarity

We designed a weighted combination technique to fuse miRNA gene functions and diseases phenotypes. The weighted combination technique can decrease the deviation of each separated similarity and balance the two different similarities. The fused miRNA similarity *MR* can be defined as Equation (7).

(7) $$\begin{array}{l} MR=\left(\gamma_{1}{MR}_{m}^{dis}+\gamma_{2}{MR}_{m}^{tar}\right)/\overset{2}{\underset{i}{\sum }}\gamma_{i} \end{array}$$

where the default value γ_*i*_ =1 indicates that the two similarities have the same weight.

### 2.5. Disease Similarity

We computed disease similarity based on the disease semantic similarity model designed by Qu et al. (2018).

#### 2.5.1. Disease Semantic Similarity Method 1

We downloaded disease semantic information from the U.S. National Library of Medicine (MeSH) (http://www.nlm.nih.gov/mesh/) and constructed a disease similarity matrix *DS* based on its Directed Acyclic Graph (DAG) (Chen et al., 2016). Suppose that *DAG*(*Dis*) = (*Dis, Set*(*Dis*), *E*(*Dis*)) represents a disease *Dis*, where *Set*(*Dis*) is a node set containing *Dis* and its ancestors, and *E*(*Dis*) is an edge set containing edges between child and parent nodes. The semantic similarity of diseases based on DAG can be computed via Equation (8):

(8) $$\begin{array}{l} D_{Dis}(d)=\{\begin{array}{cc} 1 & \text{if d }=\text{ Dis}\\ \max\{\alpha *D_{Dis}(d|d^{\prime }\in children\text{ of d})\} & \text{if d}\neq \text{Dis} \end{array} \end{array}$$

where α represents the semantic contribution factor, and the semantic contribution value of a disease to itself is 1. The semantic contribution of disease *d* to *Dis* will decrease when the distance between *d* and *Dis* increases. The semantic value of disease *Dis* can be calculated via Equation (9).

(9) $$\begin{array}{l} DS1\left(Dis\right)=\underset{d\in Set\left(Dis\right)}{\sum }D_{Dis}\left(d\right) \end{array}$$

Based on the assumption that two diseases sharing more DAGs are more similar, we computed the semantic similarity between two diseases *d*(*i*) and *d*(*j*) as

(10) $$\begin{array}{l} {SS}_{d1}\left(d\left(i\right),d\left(j\right)\right)=\frac{\underset{t\in Set\left(d\left(i\right)\right)\cap Set\left(d\left(j\right)\right)}{\sum }\left(Dis_{d\left(i\right)}\left(t\right)+Dis_{d\left(j\right)}\left(t\right)\right)}{DS1\left(d\left(i\right)\right)+DS1\left(d\left(j\right)\right)} \end{array}$$

#### 2.5.2. Disease Semantic Similarity Method 2

According to the results provided by Qu et al. (2018), different disease terms included in the same layer of a *DAG*(*D*) may appear in multiple disease *DAGs*, and furthermore, the number of their occurrences may be different. For example, for two diseases, *d*(*i*) and *d*(*j*), that appear in the same layer of the *DAG*(*D*), *d*(*i*) may appear less in disease *DAGs* than *d*(*j*). We can infer that *d*(*i*) may be more specific than *d*(*j*). Therefore, the contribution of *d*(*i*) to the semantic value of *D* should be higher than *d*(*j*). The contribution can be represented:

(11) $$\begin{array}{l} Dis_{D}2\left(d\left(i\right)\right)=-\log\left[\frac{\text{The number of DGAs including d(i)}}{\text{The number of diseases}}\right] \end{array}$$

The semantic similarity between *d*(*i*) and *d*(*j*) based on disease semantic similarity method 2 can be computed via Equation (12).

(12) $$\begin{array}{l} {SS}_{d2}\left(d\left(i\right),d\left(j\right)\right)=\frac{\underset{t\in Set\left(d\left(i\right)\right)\cap Set\left(d\left(j\right)\right)}{\sum }\left(Dis_{d\left(i\right)}2\left(t\right)+Dis_{d\left(j\right)}2\left(t\right)\right)}{DS1\left(d\left(i\right)\right)+DS1\left(d\left(j\right)\right)} \end{array}$$

#### 2.5.3. Gaussian Interaction Profile Kernel Similarity for Disease Similarity

Based on the “guilt-by-association” principle, similar diseases tend to associate with miRNAs that share more functions. Suppose that a binary vector ***ID***(*d*(*u*)) represents the interaction profile of disease *d*(*u*) associated with miRNAs: its value is set as 1 if *d*(*u*) associates with an miRNA, otherwise the value is 0. The Gaussian interaction profile kernel similarity between *d*(*i*) and *d*(*j*) is calculated as:

(13) $$\begin{array}{l} GS\left(d\left(i\right),d\left(j\right)\right)=\exp\left(-\gamma_{d}\|ID\left(d\left(i\right)\right)-ID\left(d\left(j\right)\right){\|}^{2}\right) \end{array}$$

where parameter γ_*d*_ is applied to determine the kernel bandwidth. This can be computed by standardizing a new bandwidth γ*d*′:

(14) $$\gamma_{d}=\;\frac{\gamma_{d^{\prime }}}{(\frac{1}{nd}\sum_{n=1}^{nd}∥ID(d(i)){∥}^{2})}$$

#### 2.5.4. Fused Disease Similarity

We could calculate the semantic similarity for many diseases based on their *DAGs*. However, we could not obtain *DAGs* for a few diseases and calculate their semantic similarity. Therefore, the Gaussian interaction profile kernel was used to measure the similarity for these diseases. Accordingly, we developed an integrated disease similarity measurement ***D***_***s***_ based on disease semantic similarity method 1, disease semantic similarity method 2, and the Gaussian interaction profile kernel similarity. The formulation can be computed as shown via Equation (15).

(15) $$D_{s}(d(i),d(j))=\{\begin{array}{cc} \frac{SS_{d1}(d(i),d(j))+SS_{d2}(d(i),d(j))}{2} & \text{if there is semantic similarity}\\ GS(d(i),d(j)) & \text{otherwise} \end{array}$$

## 3. RWNS

**Figure 1:**
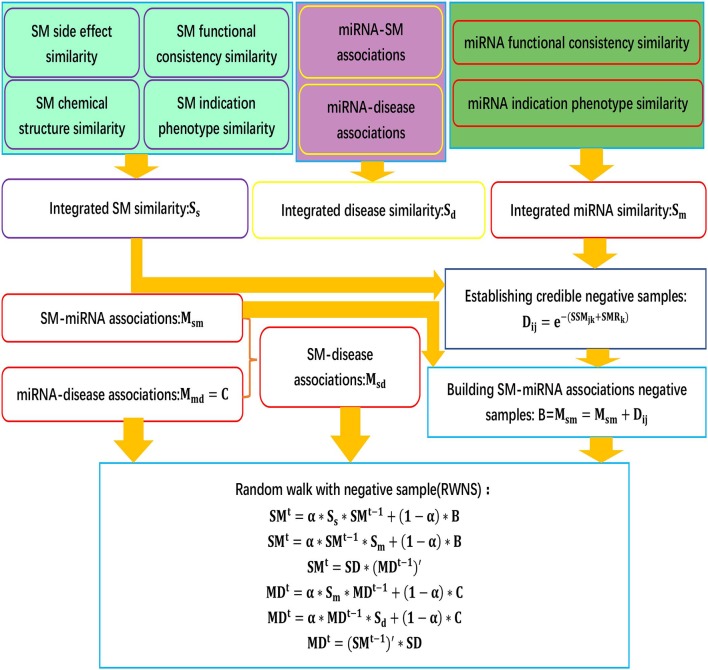
The flowchart of RWNS.

We developed an SMiR association prediction pipeline, RWNS. RWNS integrated a credible negative sample selection, random walk with restart, and diverse biological information. First, small molecule similarity, miRNA similarity, and disease similarity were computed. Highly credible negative SMiR associations were then selected based on the obtained similarity information, and random walks with restart were iteratively performed on the constructed triple-layer heterogeneous network to propagate association information and discover SMiR candidates. The details are shown in Figure 1.

### 3.1. Selecting Credible Negative SMiR Samples

High-quality negative samples can improve predictive performance. A lack of negative SMiR association samples can result in predictive bias. Consequently, it is important to integrate credible negative samples into the SMiR association prediction model. However, there is currently no public data repository that can provide negative SMiR association samples. Therefore, inspired by the negative compound-protein interaction selection method provided by Liu et al. (2015), we developed a Credible Negative Sample extraction method, CNSMiRS, to obtain high-quality negative SMiR association samples.

**Figure 2:**
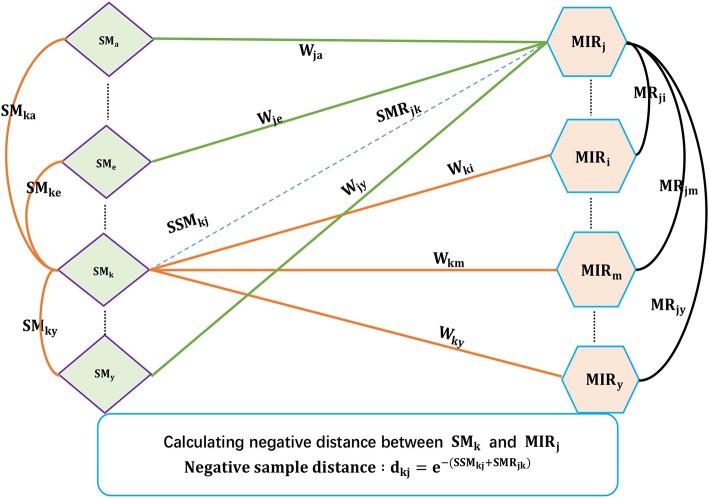
The flowchart of extracting credible negative SMiR samples.

Existing SMiR association prediction techniques are based on the assumption that similar small molecules/miRNAs are more likely to associate with miRNAs/small molecules that are more similar to the corresponding known miRNAs/small molecules. Based on the converse negative proposition of this assumption, CNSMiRS assumes that a small molecule dissimilar to every known small molecule targeting an miRNA is unlikely to associate with this miRNA. Similarly, an miRNA dissimilar to any known miRNA interacting with a small molecule is unlikely to be targeted by this small molecule. For simplicity, we represent them as the small molecule dissimilarity rule and miRNA dissimilarity rule, respectively. Both rules are used to select the most credible negative SMiR samples. This process is summarized in Algorithm 1, as can be seen in Figure 2.

**Algorithm 1:** Credible negative SMiR association sample extraction (CNSMiRS).

**Input:** Matrix ***S***_***m***_(miRNA similarity), ***S***_***s***_(small molecule similarity), ***B***(SMiR association matrix)
**Output:** CNSMiRs (Credible Negative SMiR samples)
1: *l* = the number of small molecule targeting miRNA *MR*(*k*)
2: ***w***_*kl*_ = *B*(*k, l*)
3: ***SM***_*jl*_ = ***S***_***s***_(*j, l*)
4: ***SSM***_*jkl*_(*SM*(*j*), *MR*(*k*)) = ***w***_kl_*SM_*jl*_
5: ${SSM}_{jk}\left(SM\left(j\right),MR\left(k\right)\right)=\underset{l}{\sum }{SSM}_{jkl}$.
6: *i* = the number of miRNA targeting small molecule *SM*(*j*)
7: ***w***_*ij*_ = ***B***(*i, j*)
8: ***MR***_*ik*_ = ***S***_*m*_(*i, k*)
9: ***SMR***_*kji*_(*MR*(*k*), *SM*(*j*)) = ***w***_ij_*MR_*ik*_
10: ${SMR}_{kj}\left(MR\left(k\right),SM\left(j\right)\right)=\underset{i}{\sum }{SMR}_{kji}$
11: ${\text{d}}_{kj}=e^{-\left({SSM}_{jk}+{SMR}_{kj}\right)}$
12: Rank the possible negative SMiR associations based on ***d***_*kj*_ and select those with the highest ***d***_*kj*_ as CNSMiRs.
13: Return CNSMiRs

As shown in Algorithm 1, the fused similarity for each pair of small molecules/miRNAs is firstly computed via Equations (5) and (7). Known SMiR association data are then applied to build positive sample assembly *K* in the preprocessing step. Potential negative association between small molecule *SM*(*k*) and miRNA *MR*(*j*) is denoted as (*SM*_*k*_, *MR*_*j*_, *d*_*kj*_) with *d*_*kj*_ representing the distance between small molecule *SM*(*k*) and miRNA *MR*(*j*). *d*_*kj*_ can be computed as follows.

- For any small molecule *SM*(*l*) targeting miRNA *MR*(*k*) in *K*, CNSMiRS calculates the weighted score ***SSM***_*jkl*_ = *w*_kl_*SM_*jl*_ that represents the probability of small molecule *SM*(*j*) targeting miRNA *MR*(*k*) by considering the similarity between *SM*(*j*) and *SM*(*l*). Integrating the similarity between *SM*(*j*) and each known small molecule *SM*(*l*) targeting *MR*(*j*), i.e., (*SM*_*k*_, *MR*_*j*_, *w*_*kl*_) ∈ *K*, CNSMiRS computes the associated possibility by summing up the weighed scores *SSM*_*jkl*_ related to small molecule *SM*(*l*) and thus obtains ${SSM}_{jk}=\underset{l}{\sum }{SSM}_{jkl}$.
- Similarly, CNSMiRS calculates the weighed score ***SMR***_*kji*_ = *w*_ij_*MR_*ik*_, which indicates the probability of miRNA *MR*(*k*) targeted by small molecule *SM*(*j*) by considering the similarity between *MR*(*k*) and *MR*(*i*). Integrating the similarity between *MR*(*k*) and each known miRNA *MR*(*i*)-targeted *SM*(*j*), i.e., (*MR*_*i*_, *SM*_*j*_, *w*_*ij*_) ∈ *K*, CNSMiRS computes the associated possibility by summing up the weighed scores ***SMR***_*kji*_ related to miRNA *MR*(*i*) and thus obtains ${SMR}_{kj}=\underset{i}{\sum }{SMR}_{kji}$.
- For small molecule *SM*(*j*) and miRNA *MR*(*k*), CNSMiRS calculates the distance between *SM*(*j*) and *MR*(*k*):
  (16) $$\begin{array}{l} {\text{d}}_{kj}=e^{-\left({SSM}_{jk}+{SMR}_{kj}\right)} \end{array}$$
  where ***d***_*kj*_ represents the final possibility that small molecule *SM*(*j*) does not associate with miRNA *MR*(*k*). The larger the *d*_*kj*_ is, the higher the probability of *SM*(*j*) not targeting *MR*(*k*) is.

Finally, CNSMiRS ranks negative SMiR association scores based on *d*_*kj*_ and selects those with the highest scores as negative SMiR samples.

### 3.2. Random Walk on Triple-Layer Heterogeneous Network

**Figure 3:**
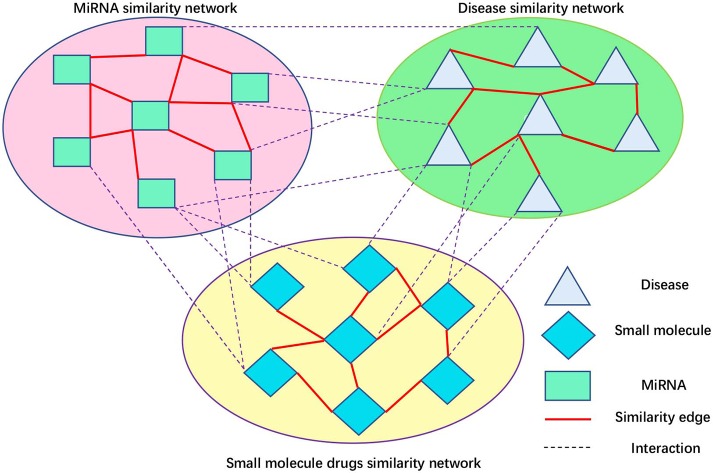
Small molecule-disease-miRNA association network.

Peng et al. (2017) developed a protein function prediction algorithm, ThrRW, based on unbalanced random walks on three biological networks. ThrRW (Peng et al., 2017) obtained a better predictive performance. Inspired by ThrRW (Peng et al., 2017), we designed an SMiR association algorithm, RWNS, based on the constructed triple-layer heterogeneous network (Figure 3). Suppose that matrix ***B***(*M* * *N*) and ***C***(*N* * *Z*) represent known SMiR and known miRNA-disease association matrix, respectively. The values of entities in these matrices are 1 (there are associations between corresponding entities) and 0 (otherwise). ***S***_***d***_(*Z* * *Z*), ***S***_***s***_(*M* * *M*), and ***S***_***m***_(*N* * *N*) are the fused disease similarity matrix, small molecule similarity matrix, and miRNA similarity matrix, respectively. ***SM***(*M* * *N*), ***MD***(*N* * *Z*), and ***SD***(*M* * *Z*) represent predicted SMiR associations, miRNA-disease associations, and small molecule-disease associations, respectively. The value of ***SM***(*i,j*) represents the probability of a small molecule *i* associating with an miRNA *j*. Similarly, ***MD***(*i,j*) represents the probability that an miRNA *i* associates with a disease *j*, and ***SD***(*i,j*) represents the probability that a small molecule *i* associates with a disease *j*.

The aim of our study was to predict possible SMiR associations according to known association information. We obtained this information by iteratively updating matrix ***SM***. The basic assumption is that the higher the similarity between the two small molecules, the higher the possibility that they interact with the same miRNA. Similarly, the higher the similarity between the two small molecules, the higher the possibility that they are associated with the same disease. RWNS developed three ways to update ***SM*** based on the assumption. Firstly, random walk steps (denoted by *l*_1_) were conducted in small molecule similarity network (***S***_***s***_) to propagate small molecule association information from their direct to level-*l*_1_ neighbors. Secondly, several random walk steps (denoted by *r*_1_) were conducted in the miRNA similarity matrix (***S***_***m***_) so that miRNAs could interact with common small molecules based on their direct to level-*r*_1_ neighbor information. Thirdly, miRNA-disease associations were transferred to small molecules through the known small molecule-disease associations (***SD***). Considering the difference between the small molecule similarity network and the miRNA similarity network, it is clear that the steps walking in these two networks are different (*l*_1_ steps in the small molecule similarity matrix, and *r*_1_ steps in the miRNA similarity matrix). Mathematically, the random walk process can be described via Equations (17–19).

(17) $$\begin{array}{lll} {SM}^{t} & = & \alpha *S_{s}*{SM}^{t-1}+\left(1-\alpha \right)*B \end{array}$$

(18) $$\begin{array}{lll} {SM}^{t} & = & \alpha *{SM}^{t-1}*Sm+\left(1-\alpha \right)*B \end{array}$$

(19) $$\begin{array}{lll} {SM}^{t} & = & SD*{\left({MD}^{t-1}\right)}^{\prime } \end{array}$$

As Equation (17) and (18) show, at each random walk step, small molecule and miRNA paths were extended (obtained by multiplying ***S***_***s***_ on the left and ***S***_***m***_ on the right), and some possible SMiR associations were thus found (achieved by updating matrix ***SM***). The parameter *t*(*t* = 1, 2, …) is the iteration steps. Matrix ***B*** as prior knowledge controls the iteration process. The parameter α ∈ [0, 1] is used to penalize longer paths and control the weight of known associations in ***B***. Because small molecules are more likely to associate with similar miRNAs, several random walks were conducted in both association networks to achieve association information of its local neighbors. Because ***S***_***s***_ and ***S***_***m***_ are different in structure and topology, two parameters (*r*_1_ and *l*_1_) were introduced to regulate maximal iteration steps in these two similarity networks. As shown in Equation (19), ***MD***^*t*−1^ stores the predicted miRNA-disease associations in the (t-1)-th step. There are some SMiR associations (stored in matrix ***SD***). Therefore, if two small molecules associate with a common disease, they may interact with a common miRNA, which is obtained by multiplying matrix ***SD*** on the left hand of matrix (***MD***^*t*−1^).

On the other hand, association matrix ***MD*** can also be updated in the manner similar to that of ***SM***. Mathematically, the random walk process can be described as

(20) $$\begin{array}{lll} {MD}^{t} & = & \alpha *Sm*{MD}^{t-1}+\left(1-\alpha \right)*C \end{array}$$

(21) $$\begin{array}{lll} {MD}^{t} & = & \alpha *{MD}^{t-1}*Sd+\left(1-\alpha \right)*C \end{array}$$

(22) $$\begin{array}{lll} {MD}^{t} & = & {\left({SM}^{t-1}\right)}^{\prime }*SD \end{array}$$

As shown in Equations (20) and (21), several random walks were conducted in ***S***_***m***_ and ***S***_***d***_, respectively. In each random walk step, some potential miRNA-disease associations (obtained by updating matrix ***MD***) could be uncovered by extending miRNA and disease paths in their corresponding networks (obtained by multiplying ***S***_***m***_ on the left and ***S***_***d***_ on the right in each iteration). Matrix ***C*** stores known miRNA-disease associations that are used to control the iteration process. Different random walk steps were conducted in two similarity networks (***S***_***m***_ and ***S***_***d***_), by performing different iteration steps (*l*_2_ steps in ***S***_***m***_ and *r*_2_ steps in ***S***_***d***_). Based on small molecule-disease associations, the predicted SMiR association information can also be transferred to diseases associated with common miRNAs by Equation (22).

In summary, RWNS integrated a credible negative sample selection, random walk on a triple-layer heterogeneous network, and various biological information into a unified framework. The details are shown in Algorithm 2. The predicted SMiR association scores based on RWNS in SM2miR2 and SM2miR1 were listed in Supplementary Material (Tables S1, S2).

**Algorithm 2:** Identifying SMiR associations based on a credible negative sample selection and random walk on triple-layer heterogeneous network(RWNS).

**Input:** Matrix ***S***_*m*_(miRNA similarity), ***S***_***d***_ (disease similarity), *S*_*s*_ (small molecule similarity), ***SD*** (small molecule-disease association matrix), ***B*** (known SMiR association matrix), ***CNSMiR*** (selected negative sample matrix), ***C*** (miRNA-disease association matrix); α, *l*_1_, *r*_1_, *l*_2_, *r*_2_.
**Output:** The predicted association score matrix ***SM*** (SMiR association matrix) and ***MD*** (miRNA-disease association matrix).
***SM***^0^ = $\frac{B}{\text{sum}\left(\text{B}\right)}$+CNSMiR;
***MD***^0^ = $\frac{C}{\text{sum}\left(\text{C}\right)}$;
**for** (*t* = 1 to **max**(*l*_1_,*r*_1_,*l*_2_,*r*_2_)) **do**
*M* = **max**(*l*_1_, *r*_1_, *l*_2_, *r*_2_)
**for** *t* = 1:*M*
*x*_1_ = 0, *x*_2_ = 0, *x*_3_ = 0
**if** *t* <= *l*_1_
${{SM}_{1}}^{t}=\alpha *S_{s}*{SM}^{t-1}+\left(1-a\right)*B$
${{SM}_{2}}^{t}=SD*{MD}^{t-1}$
*x*_1_ = 1, *x*_2_ = 1
**else**
${{SM}_{3}}^{t}=\alpha *{SM}^{t-1}*S_{m}+\left(1-a\right)*B$
*x*_3_ = 1
**end**
${SM}^{t}=\left(x_{1}*{{SM}_{1}}^{t}+x_{2}*{{SM}_{2}}^{t}+x_{3}*{{SM}_{3}}^{t}\right)/$(*x*_1_+*x*_2_+*x*_3_)
*x*_4_ = 0, *x*_5_ = 0, *x*_6_ = 0
**if** *t* < = *l*_2_
${{MD}_{1}}^{t}=\alpha *S_{m}*{MD}^{t-1}+\left(1-a\right)*C$
${{MD}_{2}}^{t}={{SM}^{t-1}}^{\prime }*SD$
*x*_4_ = 1, *x*_5_ = 1
**else**
${{MD}_{3}}^{t}=\alpha *{MD}^{t-1}*S_{d}+\left(1-a\right)*C$
*x*_6_ = 1
**end**
${MD}^{t}=\left(x_{4}*{{MD}_{1}}^{t}+x_{5}*{{MD}_{2}}^{t}+x_{6}*{{MD}_{3}}^{t}\right)/$(*x*_4_+*x*_5_+*x*_6_)
**end**
**end**
**return(SM, MD)**

## 4. Results

### 4.1. Experimental Setup and Evaluation Metrics

In this study, we performed extensive experiments to evaluate the performance of RWNS. We used leave-one-out cross validation (LOOCV) and 5-fold cross validation to compare RWNS with two state-of-the-art SMiR association methods, namely, TLHNSMMA and SMiR-NBI.

#### 4.1.1. Experimental Setup

Parameter α with range [0,1] was used to determine whether the known association state need change based on known SMiR associations (or miRNA-disease associations). In the manuscript provided by Peng et al. (2017), ThrRW obtained the best performance when the parameter α was set as 0.45. Considering the difference between ThrRW and RWNS, RWNS repeated the experiment 100 times and obtained the optimal performance when α was set as 0.4. Therefore, RWNS set α as 0.4. The four parameters *l*_1_, *r*_1_, *l*_2_, and *r*_2_ ranged from 1 to 4. Parameters *l*_1_ and *r*_1_ were used to regulate random walk steps in miRNA and small molecule similarity matrices, respectively. Parameters *l*_2_ and *r*_2_ were used to regulate random walk steps in disease and miRNA similarity matrix, respectively. The experiments were repeated 100 times. When parameters *l*_1_, *r*_1_, *l*_2_, and *r*_2_ were set as 4, 1, 1, and 1, respectively, RWNS obtained the best performance. We therefore set the five parameters as α = 0.4, *l*_1_ = 4, *r*_1_ = 1, *l*_2_ = 1, and *r*_2_ = 1. The parameters TLHNSMMA and SMiR-NBI were set as the values provided by their corresponding papers.

#### 4.1.2. Evaluation Metrics

Recall, precision, accuracy, and AUC are extensively used to evaluate different association prediction models. We used these four metrics to measure the performance of RWNS. Recall is the proportion of successfully predicted SMiR associations. Precision is the proportion of correctly predicted SMiR associations. Accuracy is the proportion of correctly predicted positive and negative SMiR associations. AUC is the area under ROC (the Receiver Operating Curve). For these four metrics, higher values indicate better prediction performance. We used these four metrics to evaluate our proposed RWNS framework. In the following two sections, experiments were performed under RWNS considering credible negative SMiR association samples. The metrics can be defined as

(23) $$\begin{array}{lll} recall & = & \frac{TP}{TP+FN} \end{array}$$

(24) $$\begin{array}{lll} precision & = & \frac{TP}{TP+FP} \end{array}$$

(25) $$\begin{array}{lll} accuracy & = & \frac{TP+TN}{TP+FP+TN+FN} \end{array}$$

where *TP*, *FP*, and *FN* are defined in Table 1.

**Table 1:** Confusion matrix of a binary classifier.

	**True class = 1**	**True class = −1**
Predicted class = 1	True positive (TP)	False positive (FP)
Predicted class = −1	False negative (FN)	True negative (TN)

### 4.2. Performance Evaluation Under LOOCV

We performed LOOCV based on the known SMiR associations in the SM2miRdatabase (Liu et al., 2012) to measure the performance of RWNS. RWNS was compared with two state-of-the-art SMiR prediction methods: SMiR-NBI (Li et al., 2016) and TLHNSMMA (Qu et al., 2018) in LOOCV. SMiR-NBI designed a network-based inference method to identify new SMiR associations. TLHNSMMA integrated SM similarity, miRNA similarity, disease similarity, experimentally verified SM-miRNA associations, and miRNA-disease associations into a heterogeneous network. The same datasets were used in these three methods. There were 664 known small molecule-miRNA associations between 831 small molecules and 541 miRNAs in dataset 1 (SM2miR1) and 664 known SMiR associations between 39 small molecules and 286 miRNAs in dataset 2 (SM2miR2). In LOOCV, each known SMiR association was chosen as the test sample in turn, and the remaining associations were used as the training samples. We conducted a series of experiments according to different negative sample selection proportion. Table 2 showed the AUC values for these three methods based on different negative sample selection proportion in two datasets. The best performance was described in boldface in each row in Table 2.

**Table 2:** The performance comparison of RWNS, TLHNSMMA, and SMiR-NBI under LOOCV on SM2miR1 and SM2miR2.

**Negative sample selection proportion (%)**	**SM2miR1**			**SM2miR2**		
	**RWNS**	**TLHNSMMA**	**SMiR-NBI**	**RWNS**	**TLHNSMMA**	**SMiR-NBI**
10	**0.9825**	0.9751	0.9015	0.7908	**0.7954**	0.7525
20	**0.9826**	0.9763	0.9016	**0.8125**	0.7614	0.7733
30	0.9825	**0.9888**	0.9016	0.7293	**0.812**	0.7619
40	0.9828	**0.9945**	0.9016	**0.8134**	0.7800	0.7851
50	**0.9826**	0.9787	0.9018	0.7586	**0.8484**	0.7837
60	0.9828	**0.9891**	0.9017	0.7726	**0.7972**	0.7851
70	0.9829	**0.9911**	0.9017	0.7980	**0.8495**	0.7829
80	0.9828	**0.9965**	0.9018	0.8835	**0.9208**	0.7866
90	**0.9827**	0.9736	0.9018	0.7908	**0.7993**	0.7885
100	0.9829	**0.9981**	0.9019	**0.8938**	0.8843	0.7993

**Figure 4:**
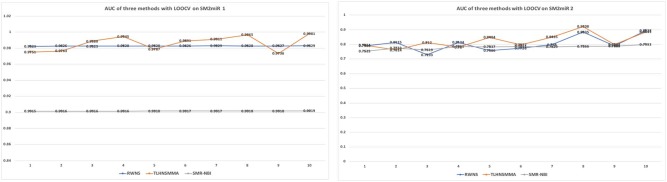
The performance comparison of RWNS, TLHNSMMA, and SMiR-NBI under LOOCV on SM2miR1 and SM2miR2.

As a result, RWNS and TLHNSMMA were superior to SMiR-NBI in two datasets. Moreover, RWNS is comparable to TLHNSMMA in LOOCV. When the negative sample selection proportion increased from 10 to 100%, the performance of the three computational models were relatively steady, and that of RWNS did not almost change in the SM2miR1 dataset. However, the AUC values slightly changed when the proportion increased in the SM2miR2 dataset, and these three methods obtained better performances when the negative sample selection proportion was 1, i.e., the number of negative samples was equal to the number of positive samples. AUCs in RWNS with dataset SM2miR1 and SM2miR2 reached 0.9829 and 0.8938, respectively. The details are shown in Figure 4.

### 4.3. Performance Evaluation Under 5-Fold Cross Validation

We performed 5-fold cross validation based on the known SMiR associations in the SM2miRdatabase (Liu et al., 2012) to evaluate the performance of RWNS. Similarly, RWNS was compared with two state-of-the-art SMiR prediction methods-SMiR-NBI (Li et al., 2016) and TLHNSMMA (Qu et al., 2018)-using 5-fold cross validation on two datasets. Tables 3, 4 showed AUC, recall, precision, and accuracy of these three methods with 5-fold cross validation based on two datasets. The best performance was described in boldface in each row in Tables 3, 4. The predicted SMiR association scores based on RWNS were shown in Tables S3, S4.

**Table 3:** The performance comparison of RWNS, TLHNSMMA, and SMiR-NBI under 5-fold cross validation in SM2miR1.

**Negative sample proportion (%)**	**AUC**			**Recall**			**Precision**			**Accuracy**		
	**RWNS**	**TLHNSMMA**	**SMiR-NBI**	**RWNS**	**TLHNSMMA**	**SMiR-NBI**	**RWNS**	**TLHNSMMA**	**SMiR-NBI**	**RWNS**	**TLHNSMMA**	**SMiR-NBI**
10	**0.9220**	0.9114	0.7123	**0.9955**	0.9472	0.7123	**1**	**1**	**1**	**0.9959**	0.9520	0.7386
20	**0.9548**	0.9487	0.7154	**0.9894**	0.9661	0.7154	0.9969	0.9962	**1**	**0.9887**	0.9688	0.7632
30	**0.9730**	0.9545	0.7228	**0.9955**	0.9718	0.7229	0.9970	0.9775	**1**	**0.9942**	0.9610	0.7873
40	**0.9780**	0.9501	0.7048	**0.9955**	0.9624	0.7049	0.9941	0.9774	**1**	**0.9925**	0.9620	0.7897
50	**0.9813**	0.9397	0.7154	**0.9925**	0.9280	0.7154	0.9896	0.9720	**1**	**0.9880**	0.9449	0.8108
60	**0.9861**	0.9364	0.7289	**0.9955**	0.9435	0.7289	0.9925	0.9563	**1**	**0.9925**	0.9366	0.8311
70	**0.9878**	0.9540	0.7154	**0.9925**	0.9604	0.7154	0.9896	0.9181	**1**	**0.9894**	0.9249	0.8332
80	**0.9899**	0.9636	0.7269	**0.9855**	0.9699	0.7169	0.9837	0.9010	**1**	**0.9883**	0.9230	0.8434
90	**0.9904**	0.9530	0.7093	**0.9955**	0.9547	0.7093	0.9895	0.8590	**1**	**0.9905**	0.8895	0.8477
100	**0.9916**	0.9638	0.7193	**0.9955**	0.9679	0.7229	0.9808	0.8821	**1**	**0.9879**	0.9163	0.8615

**Table 4:** The performance comparison of RWNS, TLHNSMMA, and SMiR-NBI under 5-fold cross validation in SM2miR2.

**Negative sample proportion (%)**	**AUC**			**Recall**			**Precision**			**Accuracy**		
	**RWNS**	**TLHNSMMA**	**SMiR-NBI**	**RWNS**	**TLHNSMMA**	**SMiR-NBI**	**RWNS**	**TLHNSMMA**	**SMiR-NBI**	**RWNS**	**TLHNSMMA**	**SMiR-NBI**
10	0.8638	**0.8963**	0.8428	**0.9549**	0.9398	0.7379	0.9784	0.9803	**0.9860**	0.9399	**0.9506**	0.7524
20	**0.9027**	0.8944	0.8876	0.9337	**0.9608**	0.7656	0.9658	0.9246	**0.9749**	0.9172	**0.9481**	0.7882
30	0.8947	0.8748	**0.9009**	0.8841	**0.8851**	0.7575	0.9515	0.8881	**0.9602**	0.8763	**0.8830**	0.7896
40	**0.9027**	0.8813	0.9498	**0.9720**	0.8926	0.7682	0.9494	0.8637	**0.9791**	**0.8756**	0.8214	0.8230
50	**0.9141**	0.8630	0.9589	0.8645	**0.8985**	0.7770	0.9384	0.8142	**0.9670**	**0.8719**	0.7950	0.8338
60	**0.9133**	0.8514	0.9560	0.8569	**0.9020**	0.8582	0.9409	0.7882	**0.9611**	**0.8771**	0.7878	0.8705
70	**0.9899**	0.9636	0.9679	**0.9855**	0.9699	0.8373	0.9136	0.8105	**0.9600**	0.8411	0.8168	**0.8843**
80	0.8856	0.9028	**0.9686**	0.7892	**0.8889**	0.8494	0.8971	0.8076	**0.9610**	0.8325	0.8206	**0.8975**
90	0.8818	0.9251	**0.9681**	0.7862	**0.9255**	0.8720	0.8917	0.8442	**0.9464**	0.8382	0.8728	**0.9086**
100	0.9048	0.9581	**0.9655**	0.7636	**0.9530**	0.8901	0.8728	**0.9304**	0.9338	0.8261	**0.9408**	0.9134

**Figure 5:**
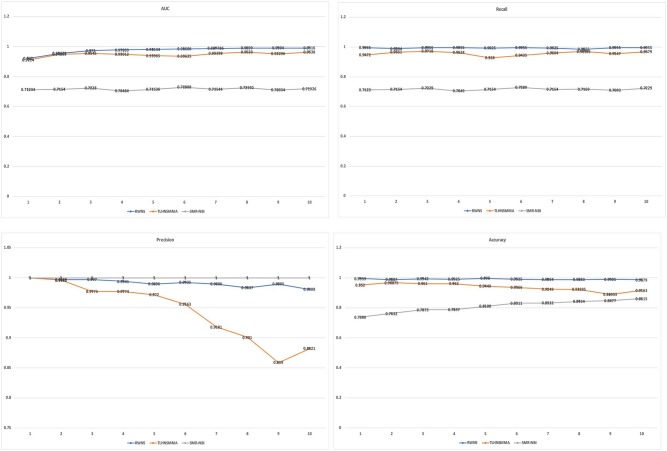
The performance comparison of RWNS, TLHNSMMA, and SMiR-NBI under 5-fold cross validation on SM2miR1.

Table 3 showed the performance of RWNS, TLHNSMMA, and SMiR-NBI based on AUC, recall, precision, and accuracy in the SM2miR1 dataset. As a result, regardless of negative sample selection proportion, RWNS obtained the best AUC, recall, and accuracy compared with SMiR-NBI and TLHNSMMA in SM2miR1. Although the performance of RWNS was not the best among these three methods according to different negative sample selection proportions, it was still fit for comparison. The results demonstrated that RWNS could better identify possible SMiR associations. Moreover, RWNS and TLHNSMMA outperformed SMiR-NBI on AUC, recall, and accuracy. SMiR-NBI obtained the highest precision when negative sample selection proportion increase from 10 to 100%. It showed that SMiR-NBI could correctly predict more SMiR associations. More importantly, RWNS achieved the highest AUC of 0.9916, recall of 0.9955 and accuracy of 0.9879 when the negative sample selection proportion was 100%. Based on the comprehensive measurement of the experimental results, RWNS gave the optimal performance, followed by TLHNSMMA and SMiR-NBI. The details are shown in Figure 5.

**Figure 6:**
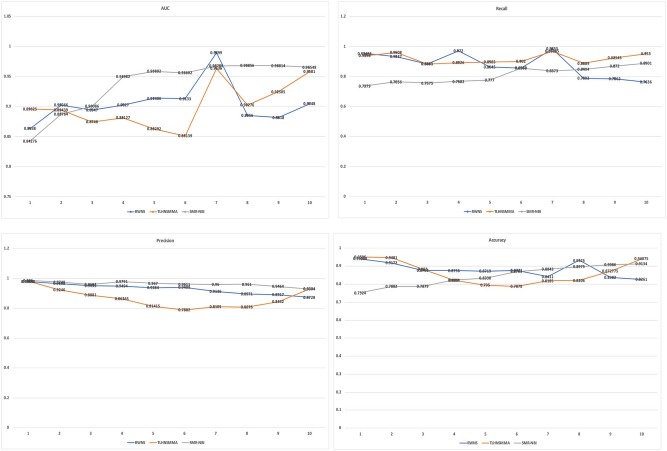
The performance comparison of RWNS, TLHNSMMA, and SMiR-NBI under 5-fold cross validation on SM2miR2.

Table 4 showed the performance of RWNS, TLHNSMMA, and SMiR-NBI based on AUC, recall, precision, and accuracy in the SM2miR2 dataset. None of these three methods outperformed the other two methods when the negative sample selection proportion changed, and this may be caused by different data structures. Moreover, when the negative sample selection proportion was 0.7, RWNS obtained a better performance, and AUC, recall, precision, and accuracy were 0.9899, 0.9855, 0.9136, and 0.8325, respectively. The details are shown in Figure 6.

### 4.4. Performance Comparison Considering CNSMiRS or Not

In this section, we analyzed the effect of credible negative sample selection on predictive performance. We compared RWNS+CNSMiRS (RWNS considering negative sample selection) with RWNS-CNSMiRS (RWNS not considering negative sample selection). The results are shown in Table 5. As shown in Table 5, RWNS+CNSMiRS resulted in a better performance than RWNS-CNSMiRS in two datasets. In the SM2miR1 dataset, RWNS+CNSMiRS obtained the AUC value of 0.9916, while RWNS-CNSMiRS obtained 0.9875. In the SM2miR2 dataset, RWNS+CNSMiRS obtained the AUC value of 0.9899, while RWNS-CNSMiRS obtained 0.7865. The results suggested that credible negative SMiR association samples may help improve predictive performance. The best performance was described in boldface in each row in Table 5. The predicted negative SMiR association scores based on CNSMiRS were shown in Tables S3, S4.

**Table 5:** Performance comparison considering CNSMiRS or not.

	**SM2miR1**	**SM2miR2**
RWNS+CNSMiRS	**0.9916**	**0.9899**
RWNS-CNSMiRS	0.9875	0.7865

There were 449,571 and 11,154 small molecule-miRNA pairs in SM2miR1 and SM2miR2, respectively. However, there were only 664 experimentally validated SMiR associations in two datasets. In the SM2miR1 dataset, unobserved samples were more than that of SM2miR2, and thus selected negative samples were more than of SM2miR2. More negative samples may have helped improve predictive accuracy. Therefore, RWNS+CNSMiRS exhibited a better performance in the SM2miR1 dataset than SM2miR2.

### 4.5. Case Study

In this study, we extracted the top 50 SMiR associations with the highest scores and validated these associations from the published references in the PubMed database by retrieving the related documents. The details are shown in Table 6. Among the predicted top 10, 20, and 50 associations with the highest scores, there are 9, 17, and 37 SMiR associations that can be validated by the other documents, respectively. That is to say, among the predicted 10, 20, and 50 associations with the highest scores, 90, 85, and 74% associations can be confirmed by the published references in the PubMed database, respectively. The results demonstrated that RWNS can effectively identify SMiR association candidates. For TLHNSMMA, among the predicted top 10, 20, and 50 SMiR associations with the highest scores, there are only 2, 4, and 14 associations that can be validated by the published documents, respectively. There are three SMiR associations that can be simultaneously identified by RWNS and TLHNSMMA. The results suggest that RWNS may more accurately find possible SMiR associations.

**Table 6:** The predicted top 50 SMiR associations with the highest scores.

**Number**	**Small molecule**	**miRNA**	**Confirmed**	**Number**	**Small molecule**	**miRNA**	**Confirmed**
1	CID:60750	hsa-mir-21	24331411	26	CID:3385	hsa-mir-17	21516486
2	CID:31703	hsa-mir-21	19412672	27	CID:3229	hsa-mir-155	No
3	CID:36462	hsa-mir-21	17554199	28	CID:5331	hsa-mir-17	No
4	CID:3229	hsa-mir-21	No	29	CID:9444	hsa-mir-125b-1	No
5	CID:3385	hsa-mir-21	22382630	30	CID:451668	hsa-mir-125b-1	No
6	CID:451668	hsa-mir-21	16530703	31	CID:60953	hsa-mir-125b-1	20370587
7	CID:5757	hsa-mir-21	25179838	32	CID:9444	hsa-mir-21	25939322
8	CID:10635	hsa-mir-21	20945501	33	CID:3229	hsa-mir-125b-1	No
9	CID:5288826	hsa-mir-21	30680008	34	CID:36462	hsa-mir-125b-1	27174811
10	CID:3121	hsa-mir-21	24126255	35	CID:9444	hsa-mir-20a	No
11	CID:60750	hsa-mir-155	22399498	36	CID:451668	hsa-mir-20a	17660710
12	CID:451668	hsa-mir-155	No	37	CID:5288826	hsa-mir-20a	28070858
13	CID:5288826	hsa-mir-155	26072390	38	CID:3385	hsa-mir-20a	25960225
14	CID:5311	hsa-mir-155	19513533	39	CID:448537	hsa-mir-20a	28131841
15	CID:448537	hsa-mir-21	27521771	40	CID:451668	hsa-mir-145	24283360
16	CID:5311	hsa-mir-21	27557899	41	CID:448537	hsa-mir-155	No
17	CID:5288826	hsa-mir-146a	30827946	42	CID:9444	hsa-mir-145	26440147
18	CID:5757	hsa-mir-146a	18634034	43	CID:5311	hsa-mir-34a	23759592
19	CID:3229	hsa-mir-146a	No	44	CID:448537	hsa-mir-34a	27659519
20	CID:9444	hsa-mir-17	22072491	45	CID:451668	hsa-mir-34a	21323860
21	CID:451668	hsa-mir-17	17660710	46	CID:9444	hsa-mir-125b-2	No
22	CID:448537	hsa-mir-17	No	47	CID:36314	hsa-mir-21	24137413
23	CID:3385	hsa-mir-155	21516486	48	CID:451668	hsa-mir-125b-2	28105425
24	CID:5757	hsa-mir-155	26771440	49	CID:31703	hsa-mir-145	21217773
25	CID:5757	hsa-mir-17	24658544	50	CID:60953	hsa-mir-125b-2	No

Among the predicted top 10 SMiR associations, 10 different small molecules were associated with the same miRNA (hsa-mir-21). Mir-21 is a kind of non-protein-coding RNA and can regulate the expression of related target genes to control tumorigenic processes (Esteller, 2011). This clinical study has shown that overexpression of mir-21 plays an essential role in primary breast cancer, lung cancer (Bica-Pop et al., 2018), gastric cancer (Zhang et al., 2008; Tsujiura et al., 2010), and normal adjacent tumor tissues (Negrini and Calin, 2008; Markou et al., 2013). Higher expression of mir-21 is related to lower overall survival rates of patients (Teixeira et al., 2014). The nine known small molecules are confirmed to associate with mir-21 and are used to control cancer initiation and progression (Krichevsky and Gabriely, 2009). The remaining small molecule (CID:3229) is predicted to interact with mir-21. Therefore, we have inferred that small molecule (CID:3229) probably interact with mir-21 and can be applied to control cancer initiation and progression.

Among the predicted top 20 SMiR associations, we discovered new interactions related to mir-155 and mir-146a. Mir-155 can control and regulate various physiological and pathological processes (Friedman et al., 2009). Some clinical studies have found that mir-155 is overexpressed in pancreatic juice samples from pancreatic cancer patients, and mir-155 may control pathological processes related to pancreatic cancer (Sadakari et al., 2010).

Among the predicted results, gemcitabine (CID:60750), doxorubicin (CID:31703), etoposide (CID:36462), and fluoracil (CID:3385) are small molecules associated with mir-21. They have similar functions and can destroy DNA molecular structures to inhibit DNA synthesis, reconstruct DNA topological structures, and prevent cell entry into the mitotic phase of cell division and thus lead to cell death. The process arrests tumor growth and result in apoptosis. Associations between these four small molecules and mir-21 are ranked as one, two, three, and five, respectively. The functions of enoxacin (CID:3229) are similar to the above small molecules. It can inhibit DNA topoisomerase type II (atp-hydrolyzing) activity. DNA topoisomerase type II plays an essential role in relaxing supercoiled DNA. Therefore, we inferred that enoxacin may be associated with mir-21.

Moreover, gemcitabine (CID:60750) and vorinostat (CID:5311) can inhibit the process of cell division and thus lead to cell death. The process arrests tumor growth and result in apoptosis. Decitabine (CID:451668) can be incorporated into DNA during replication and RNA during transcription. The process can regulate way of proteins binding to the RNA/DNA substrate and control the process of cell division. Decitabine (CID:451668), gemcitabine (CID:60750), and vorinostat (CID:5311) have similar pharmacodynamics functions. Gemcitabine (CID:60750) and vorinostat (CID:5311) associate with mir-155. Therefore, we have inferred that decitabine (CID:451668) may interact with miRNA-155.

## 5. Conclusion and Further Research

The overexpression of miRNA can result in various complex human diseases. Identifying possible SMiR associations help genomic pharmacy studies. However, experimental methods for SMiR association prediction are still expensive, time-consuming, and laborious processes. Many computational methods have therefore been developed to address this problem.

In this study, we developed an SMiR association prediction method, RWNS, integrating various biological information, credible negative sample selection, and random walk on triple-layer heterogeneous network into a unified framework. We compared the performance of RWNS with TLHNSMMA and SMiR-NBI based on AUC, recall, precision, and accuracy. The results showed that RWNS obtained better performance and could effectively predict possible SMiR associations. Moreover, we analyzed the predicted top 50 SMiR associations with the highest scores and found that enoxacin and decitabine may be associated with mir-21 and mir-155, respectively. Therefore, RWNS could be an effective tool for SMiR association prediction.

Biological information help find SMiR candidates in a more accurately way. RWNS fused different biological information related to small molecules and miRNAs. However, it may be improved by integrating more data, for example, functional associations between microRNAs and long non-coding RNAs (Zhang et al., 2018b). More importantly, how to integrate these data is still an ongoing challenge. In the future, we will further consider deep learning-based models to better integrate diverse biological data and improve predictive performances. Finally, the linear neighborhood propagation method (Zhang et al., 2018a, 2019c) may be efficiently applied to SMiR association prediction.

## Data Availability Statement

The authors declare that the data supporting the findings of this study are available within the article/Supplementary Material.

## Author Contributions

FL, LP, GT, JY, and LZ developed the negative sample selection method. FL, LP, GT, and HC wrote the paper, and JY, QH, and XL revised the original draft. All authors read and approved the final manuscript.

### Conflict of Interest

GT and JY were employed by the company Geneis (Beijing) Co. Ltd. The remaining authors declare that the research was conducted in the absence of any commercial or financial relationships that could be construed as a potential conflict of interest.

## Supplementary Material

Table S1 The predicted SMiR association scores based on RWNS in SM2miR1.

Table S2 The predicted SMiR association scores based on RWNS in SM2miR2.

Table S3 The predicted negative SMiR association scores based on CNSMiR in SM2miR1.

Table S4 The predicted negative SMiR association scores based on CNSMiR in SM2miR2.
